# The best objective response of target lesions and the incidence of treatment-related hypertension are associated with the survival of patients with metastatic renal cell carcinoma treated with sunitinib: a Japanese retrospective study

**DOI:** 10.1186/s13104-016-1895-8

**Published:** 2016-02-09

**Authors:** Makito Miyake, Masaomi Kuwada, Shunta Hori, Yosuke Morizawa, Yoshihiro Tatsumi, Satoshi Anai, Yukinari Hosokawa, Yoshiki Hayashi, Atsushi Tomioka, Takeshi Otani, Kenji Otsuka, Yoshinori Nakagawa, Yasushi Nakai, Shoji Samma, Nobumichi Tanaka, Kiyohide Fujimoto

**Affiliations:** Department of Urology, Nara Medical University, 840 Shijo-cho, Nara, 634-8522 Japan; Department of Pathology, Nara Medical University, Nara, Japan; Department of Urology, Tane General Hospital, Osaka, Japan; Department of Urology, Matsuzaka Chuo General Hospital, Mie, Japan; Department of Urology, Yamato Takada Municipal Hospital, Nara, Japan; Department of Urology, Nara Prefecture General Medical Center, Nara, Japan

**Keywords:** Renal cell carcinoma, Sunitinib, Survival, RECIST

## Abstract

**Background:**

The aim of this study is to investigate the prognostic relevance of the best objective response of metastatic target lesions during sunitinib treatment in patients with metastatic renal cell carcinoma.

**Methods:**

Radiographic analysis of the best objective response according to the Response Evaluation Criteria in Solid Tumors was assessed in 50 patients. Clinicopathological characteristics including the Heng risk classification and sunitinib-related adverse reactions were compared among four patient subgroups [complete response or partial response (CR/PR), stable disease (SD), progressive disease (PD), and those without treatment evaluation (NE)]. Kaplan–Meier and Cox proportional regression analyses of progression-free survival and overall survival were performed to identify prognostic variables.

**Results:**

The best objective response was CR/PR in 12 (24 %) patients, SD in 22 (44 %), PD in 6 (12 %), and NE in 10 (20 %). The incidence of hypertension and hypothyroidism was associated with a better objective response. Progression-free survival was 15.0, 9.2, 6.8, and 2.2 months in the CR/PR, SD, PD, and NE groups, respectively (*P* = 0.0004, log-rank test), while the corresponding median overall survival was 59.7, 24.2, 17.1, and 18.1 months, respectively (*P* = 0.007). Multivariate analysis revealed that hazard ratios for risk of death of the SD, PD, and NE groups were 4.51 (*P* = 0.06), 7.93 (*P* = 0.02), and 4.88 (*P* = 0.04), respectively, as compared to the CR/PR group.

**Conclusions:**

Our findings suggested that the best objective response of target lesions was a prognostic marker for both progression-free survival and overall survival in sunitinib treatment. Furthermore, the incidence of sunitinib-induced hypertension was associated with a longer progression-free survival.

## Background

For several decades, treatment for patients with advanced and metastatic renal cell carcinoma (mRCC) has relied on systemic immunological cytokine therapies, such as interferon alpha and interleukin-2 [1–3]. Substantial advances in the understanding of the molecular biology of renal cell carcinoma have led to the development of several anti-angiogenic agents, including tyrosine kinase inhibitors (TKIs) and mammalian targeted of rapamycin inhibitors (mTORIs), which are currently available for the treatment for mRCC [4–7].

Previous prognostic models often account for different clinical and laboratory values at baseline, and successfully capture the clinical behavior and biological characteristics of mRCC. However, recent studies have investigated the clinical relevance of tumor size remission induced by targeted therapies such as TKIs and mTORIs [8–13]. In one of the largest studies, a subset of 468 patients undergoing phase I trials were evaluated; a linear association was observed between survival and tumor shrinkage, as assessed by categorical response evaluation [10]. Another study identified tumor shrinkage of ≥10 % within the first 12 weeks of targeted therapy as a prognostic parameter for longer survival [12]. In contrast, a recent study reported that tumor remission [complete response (CR) or partial response (PR)] was not associated with superior overall survival (OS) as compared to stable disease (SD) in their cohort [13], thus the correlation of tumor response to therapy is a complex entity effected by numerous factors; drug absorption, drug distribution, drug metabolism and the ability to sustain serum drug levels (i.e., not miss doses due to side effects).

Sunitinib (SU011248; Sutent®, Pfizer Inc.) is a multi-targeted TKI available since 2006 in European countries, since 2007 in the United States, and since 2008 in Japan. It has been approved worldwide as the first-line treatment for selected clear-cell mRCC patients, with a reportedly significant objective response rate of up to 47 % [5, 7]. Nevertheless, the majority of patients develop treatment resistance, whereas a number of them have no clinical benefits from sunitinib therapy, thus requiring further sequential therapy. Therefore, the identification of clinically applicable prognostic and predictive markers for longer progression-free survival (PFS) and OS is essential for the improvement of mRCC patients’ outcomes.

Although some evidences regarding the association of tumor remission by sunitinib and superior outcomes has been reported in Europe, there is a lack of such data on Asian cohorts, particularly Japanese patients. The aim of this study was to investigate the prognostic value of tumor remission induced by sunitinib and its influence on the PFS and OS in a Japanese multicenter cohort.

## Methods

### Patients and data collection

The medical records of patients with mRCC treated with sunitinib at five institutions between May 2008 and July 2013 were retrospectively reviewed. This study was approved by The Ethics Committee of Nara Medical University. The analysis was performed in accordance with the Declaration of Helsinki (64th World Medical Association General Assembly, Fortaleza, Brazil, in October 2013). No restrictions on histological subtype were applied.

There were no restrictions on the number of prior therapies, including cytokine treatment and sorafenib, but no patients received prior therapy with axitinib, pazopanib, or mTORIs. Patients were required to have adequate baseline organ functions, including renal function defined by a serum creatinine level of <2.0 mg/dL, liver function defined by a serum bilirubin level of ≤1.5× upper limit of normal (ULN) and serum transaminases activity of ≤2.5× ULN; and bone marrow function defined by an absolute neutrophil count of ≥1500/μL, platelet count of ≥10 × 10^4^/μL, and hemoglobin level of >9.0 g/L. Fifty eligible patients were classified into three groups according to their Heng risk score [14]. Of these, 21 (42 %) received cytokine therapy, and 6 (12 %) received sorafenib prior to the induction of sunitinib. The cut-off values for baseline serum C-reactive protein (CRP) and sodium levels were 0.3 mg/mL and 135 mmol/L, respectively, as commonly reported in other studies [15, 16].

### Sunitinib treatment and follow-up

Each cycle of sunitinib consisted of four consecutive weeks of treatment at 25–50 mg daily, followed by a 2-week break. In case of significant toxicity, dose reduction was applied to avoid early treatment termination and improve the patients’ quality of life. Sunitinib doses were reduced from 50 to 37.5 mg, and further to 25 mg if necessary. Patient follow-up and laboratory examination were routinely performed on days 1, 15, and 29 of the first treatment cycle, and at least every 14 days during sunitinib treatment thereafter. Adverse reactions were graded according to the Common Terminology Criteria for Adverse Events version 4.0. Radiographic examination including computed tomography (CT) and magnetic resonance imaging was performed every 1–3 cycles to evaluate treatment response in accordance with the Response Evaluation Criteria in Solid Tumors (RECIST) version 1.1 [17]. Patients were categorized into four different groups according to their calculated best objective responses of target metastatic lesions as follows: CR/PR, SD, progressive disease (PD), and those without treatment evaluation, not evaluated (NE).

Diagnosis and treatment of hypertension was carried out as follows. The patients were asked to measure blood pressure (BP) at least twice a day during sunitinib treatment. BP was measured by the nurses at the clinic visit. The mean value of systolic and/or diastolic BP of home and clinic measurements was calculated. Principally, ≥140 and/or ≥90 mmHg was recognized as hypertension requiring antihypertensive treatment. All efforts were made to control BP below 140/90 mmHg.

### Statistical analysis

PFS was defined as the time between treatment initiation and progression. Progression was defined according to the RECIST radiographic criteria and clinical criteria that prevented treatment continuation. OS was defined as the time between sunitinib therapy initiation and the date of death. Cases with no events at the time of analysis were censored from the statistical analyses.

PFS and OS curves were generated using the Kaplan–Meier method, and compared by using the log-rank test or log-rank test for trend for each prognostic variable. Multivariate analysis was employed to identify independent prognostic variables using a stepwise Cox proportional hazards regression model. Variables influencing survival according to univariate analysis were included in the multivariate analysis. IBM SPSS version 21 (SPSS Inc., Chicago, IL) and PRISM software version 5.00 (GraphPad Software, Inc., San Diego, CA) were used for statistical analyses and data plotting, respectively. A *P* value of <0.05 was considered statistically significant.

## Results

### Patient characteristics

A total of 50 patients with a measurable response to sunitinib treatment were included in the study (Table 1). Of these patients, 4 (8 %) achieved CR as their best objective response; 8 (16 %) achieved PR; 22 (44 %) achieved SD; 6 (12 %) experienced PD, and 10 (20 %) were NE. Of the 10 NE patients, 5 discontinued sunitinib owing to severe adverse effects; 4 patients exhibited PD before radiographic evaluation, and 1 died of unknown cause 1 week after sunitinib initiation. The clinicopathological variables were compared among the patient groups (Table 1). Overall, 88 % of the patients underwent nephrectomy prior to sunitinib therapy. Patients with poor Eastern Cooperative Oncology Group performance status (ECOG-PS, 2 or 3) tended to have a poor response of targeted lesions or required treatment discontinuation as compared to those with a good ECOG-PS (0 or 1). In addition, patients with a poorer Heng risk score had a significantly worse response of targeted lesions (*P* = 0.016), and most of them (7 out 11) were categorized in the PD and NE groups. No further significant differences in the clinicopathological variables were observed among the four patient groups.

**Table 1:** Demographic and clinical parameters of 50 patients with mRCC treated with sunitinib

Variables	All cases (n = 50)	CR/PR (n = 12)	SD (n = 22)	PD (n = 6)	NE (n = 10)	*P* value
Age [median (IQR)]	64 (58–71)	63 (56–77)	63 (57–67)	61 (56–66)	70 (64–76)	0.16^†^
Follow-up months [median (IQR)]	20 (10–25)	21 (15–25)	22 (13–30)	12 (6–23)	18 (7–25)	0.38^†^
Gender						0.51^‡^
Male	40 (80 %)	10 (83 %)	17 (77 %)	6 (100 %)	7 (70 %)	
Female	10 (20 %)	2 (17 %)	5 (23 %)	0 (0 %)	3 (30 %)	
Prior nephrectomy						0.95^‡^
Yes	44 (88 %)	11 (92 %)	19 (86 %)	5 (83 %)	9 (90 %)	
No	6 (12 %)	1 (8 %)	3 (14 %)	1 (17 %)	1 (10 %)	
Tumor histology						0.41^‡^
Clear cell	40 (80 %)	11 (92 %)	15 (68 %)	5 (83 %)	9 (90 %)	
Non-clear cell	4 (8 %)	0 (0 %)	4 (18 %)	0 (0 %)	0 (0 %)	
Unknown	6 (12 %)	2 (17 %)	3 (14 %)	1 (17 %)	1 (10 %)	
Concomitant sarcomatoid variant	2 (4 %)	0 (0 %)	0 (0 %)	1 (17 %)	1 (10 %)	
ECOG-PS						0.06^‡^
0/1	41 (82 %)	11 (92 %)	19 (86 %)	5 (83 %)	5 (50 %)	
2/3	9 (18 %)	1 (8 %)	3 (14 %)	1 (17 %)	5 (50 %)	
Metastatic sites						0.24^‡^
Lung	28 (56 %)	7 (58 %)	12 (55 %)	5 (83 %)	4 (40 %)	
Lymph nodes	12 (24 %)	3 (25 %)	8 (36 %)	2 (33 %)	1 (10 %)	
Bone	19 (38 %)	2 (17 %)	10 (45 %)	0 (0 %)	5 (50 %)	
Liver	4 (8 %)	1 (8 %)	1 (5 %)	1 (17 %)	1 (10 %)	
Pancreas	2 (4 %)	2 (17 %)	0 (0 %)	0 (0 %)	0 (0 %)	
Heng risk group						0.016^‡^
Favorable	10 (20 %)	3 (25 %)	5 (23 %)	1 (17 %)	1 (10 %)	
Intermediate	29 (58 %)	8 (67 %)	14 (63 %)	3 (50 %)	4 (40 %)	
Poor	11 (22 %)	1 (8 %)	3 (14 %)	2 (33 %)	5 (50 %)	
Serum CRP (mg/dL) (mean ± SEM)	2.70 ± 0.61	1.35 ± 0.78	2.79 ± 0.90	2.62 ± 1.43	4.18 ± 1.98	0.41^†^
Serum sodium (mmol/L) (mean ± SEM)	139.4 ± 0.38	139.8 ± 0.59	140.3 ± 0.43	138.7 ± 1.26	137.4 ± 1.08	0.09^†^

*IQR* interquartile range; *ECOG-PS* Eastern Cooperative Oncology Group-perfomance status; *CRP* C-reactive protein; *CR* complete response; *PR* pertial response; *SD* stable disease; *PD* progressive disease; *NE* not evaluated; *SEM* standard error of mean

^†^Kruskal–Wallis test; ^‡^ Chi square test or Fisher’s exact test

### The association between the best objective response and survival

**Fig. 1:**
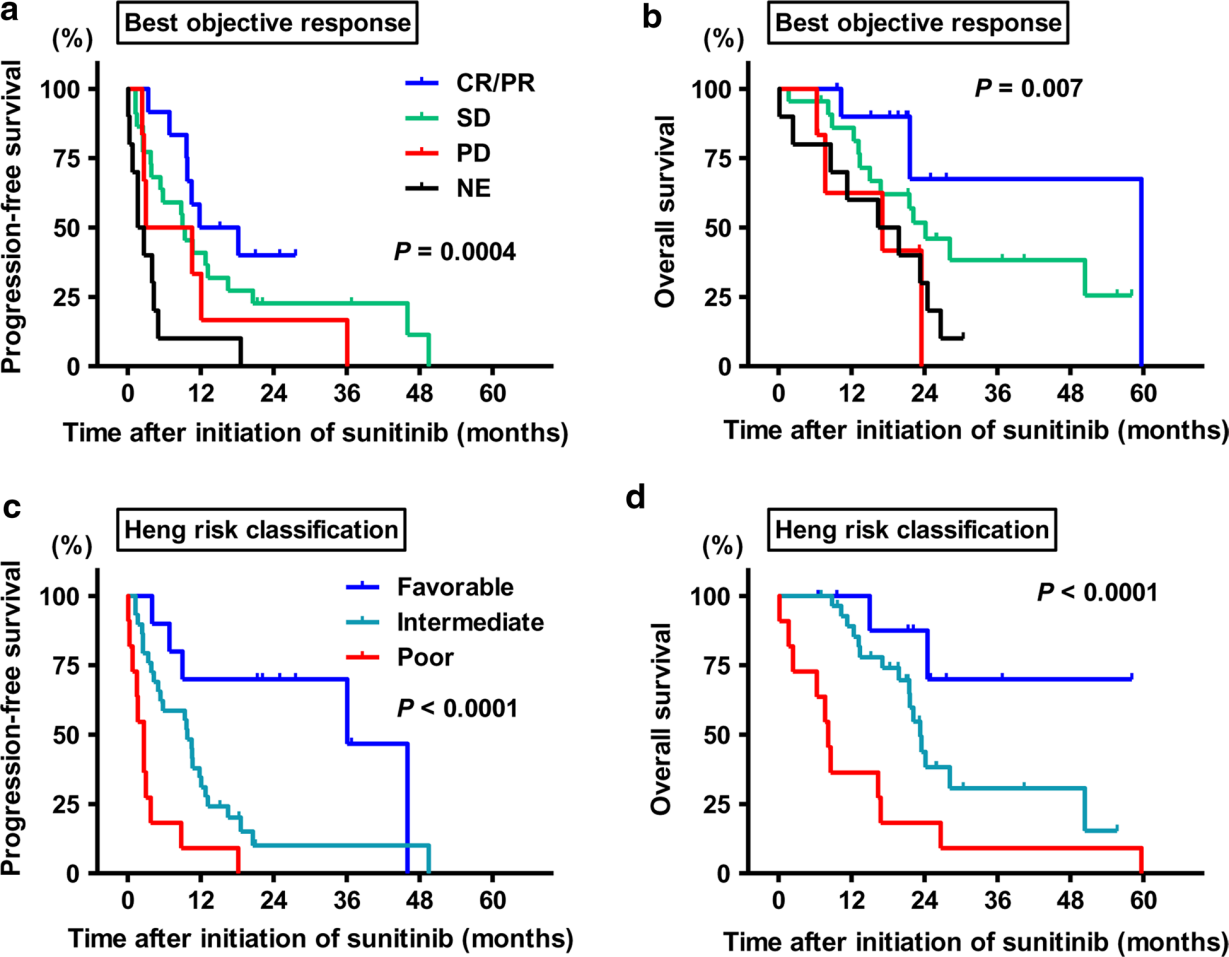
Kaplan–Meier analysis of progression-free survival (*left panels*) and overall survival (*right panels*). Kaplan–Meier estimates of survivals according to the best objective response of metastatic target sites (**a**, **b**), Heng risk score (**c**, **d**). The time to the events is given in months

The median overall follow-up period was 20 months. Table 2 presents the association between the best objective response of target lesions and the median PFS and OS after sunitinib initiation. Kaplan–Meier analysis revealed that the PFS and OS at 1 year were 35 and 81 %, respectively, whereas the corresponding values at 3 years were 15 and 35 %, respectively. Patients treated with sunitinib reached a median PFS of 8.9 months and a median OS of 23.5 months. Stratification according to the best objective response showed that the median PFS was 15.0 months among CR patients, 9.2 months for SD patients, 6.8 months for those with PD, and 2.2 months among NE patients, while the corresponding median OS was 59.7, 24.2, 17.1, and 18.1 months, respectively. There was a clear trend in the association between better tumor response and longer PFS and OS (Fig. 1a, b).

**Table 2:** Association of survival after sunitinib therapy and the best objective response on target lesions

Best objective response on target lesions	Progression-free survival^†^			Overall survival^†^		
	Median survial (months)	1-year (%)	3-years (%)	Median survial (months)	1-year (%)	3-years (%)
All cases	8.9	35	15	23.5	81	35
CR/PR	15.0	50	40	59.7	90	68
SD	9.2	41	23	24.2	81	38
PD	6.8	27	17	17.1	63	0
NE	2.2	10	0	18.1	60	10

*IQR* interquaterile range; *CR* complete response; *PR* pertial response; *SD* stable disease; *PD* progressive disease; *NE* not evaluated

^†^Estimated with Kaplan–Meier method

### Adverse reactions induced by sunitinib

**Fig. 2:**
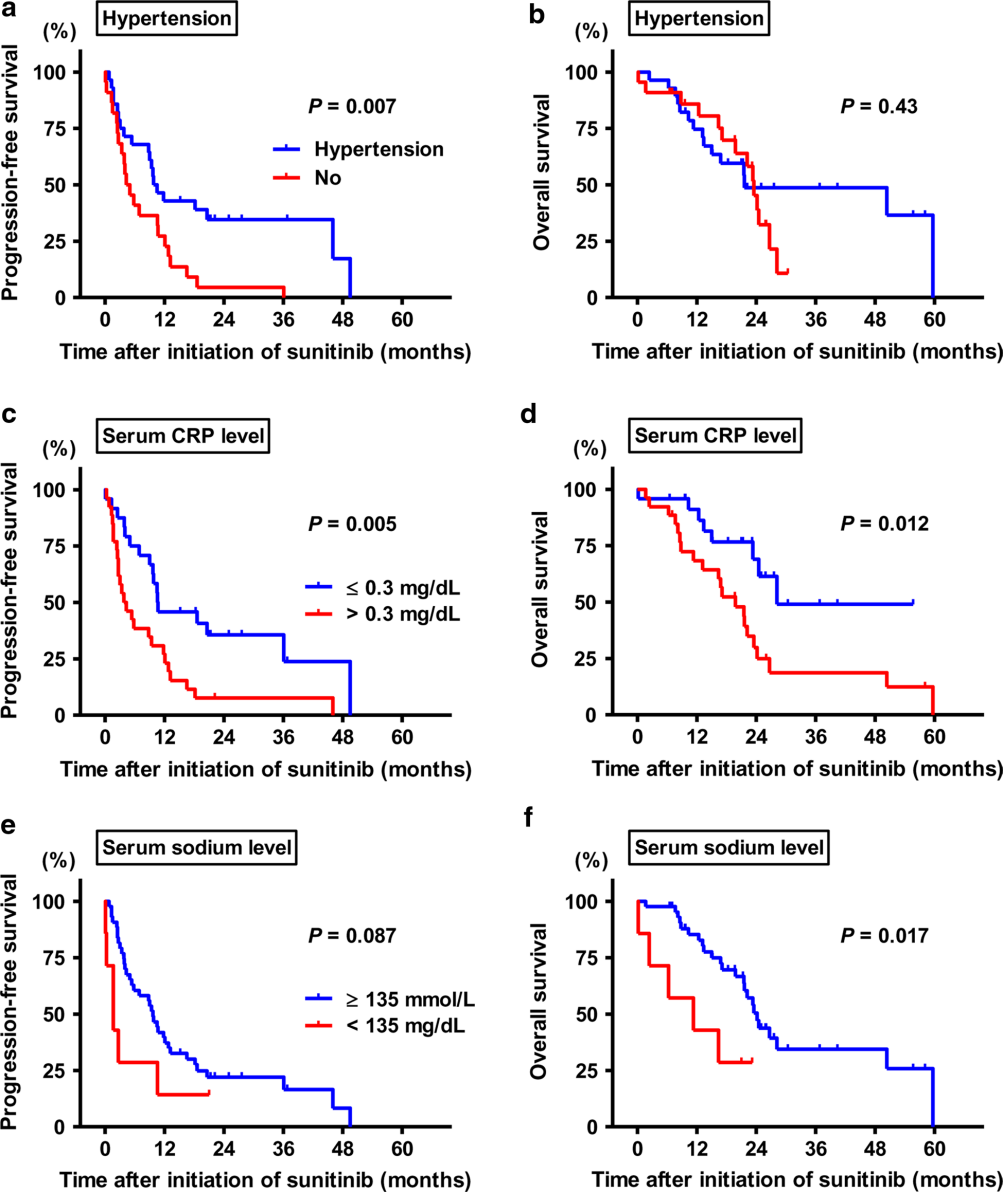
Kaplan–Meier analysis of progression-free survival (*left panels*) and overall survival (*right panels*). Kaplan–Meier estimates of survivals according to the incidence of sunitinib-related hypertension (**a**, **b**), serum level of CRP (**c**, **d**) and sodium (**e**, **f**) at baseline. The time to the events is given in months

All patients experienced some kinds of adverse reactions during sunitinib treatment (Table 3). The most frequently observed adverse reactions were hematologic toxicity, including anemia and decrease in neutrophils, lymphocytes, and platelets. Grade 3 adverse effects were observed in 30 % (15 out of 50 cases) of all patients, and severe thrombocytopenia accounted for 80 % (12 out of 15 cases) of these events. No grade 4 or 5 adverse effects were observed. Hypertension, hypothyroidism, fatigue, diarrhea, oral mucositis, and hand-foot syndrome were commonly observed non-hematologic toxicity. There was a significant association between better tumor response and the incidence of hypertension and hypothyroidism (*P* = 0.026 and *P* = 0.043 by using the Fisher’s exact test, respectively). Kaplan–Meier analysis revealed that the incidence of hypertension was associated with a longer PFS (*P* = 0.007, Fig. 2a) but not OS (*P* = 0.43, Fig. 2b), while neither the incidence of hypothyroidism nor that of grade 3 adverse reactions had any prognostic value (data not shown).

**Table 3:** Comparison of adverse events by the best objective response on target lesions

Adverse events	All grades (%)					Grades 3/4 (%)				
	All cases (n = 50)	CR/PR (n = 12)	SD (n = 22)	PD (n = 6)	NE (n = 10)	All cases (n = 50)	CR/PR (n = 12)	SD (n = 22)	PD (n = 6)	NE (n = 10)
Any	100	100	100	100	100	30	25	27	50	30
*Hematologic*										
Anemia	58	58	54	67	60	4	0	0	17	10
Neutorophils decreased	44	50	46	67	20	6	0	5	33	0
Lymphocyte decreased	36	42	36	50	20	6	8	5	0	10
Platelet decreased	58	67	50	83	50	24	17	27	50	10
Creatinine increased	38	33	41	33	40	0	–	–	–	–
*Non-hematologic*										
Hypertension*	56	84	59	33	30	2	0	5	0	0
Hypothyroidism*	46	42	59	33	30	0	–	–	–	–
Fatigue	36	33	32	50	40	0	–	–	–	–
Diarrhea	30	33	36	0	30	0	–	–	–	–
Mucositis oral	28	17	46	17	10	0	–	–	–	–
Hand-foot syndrome	20	25	27	17	0	0	–	–	–	–
Fever up	18	25	9	33	20	0	–	–	–	–
Hemorrhage	16	8	23	17	10	0	–	–	–	–
Heart failure	4	8	5	0	0	0	–	–	–	–

All the statistical analysis is performed with Fisher’s exact test, * *P* < 0.05

*CR* complete response; *PR* pertial response; *SD* stable disease; *PD* progressive disease; *NE* not evaluable

### Multivariate analyses of prognostic parameters for PFS and OS

To explore the prognostic parameters for PFS and OS in mRCC patients treated with sunitinib, we performed univariate analysis followed by multivariate analyses. Univariate analysis identified the best objective response (*P* = 0.0004, Fig. 1a), Heng risk score (*P* < 0.0001, Fig. 1c), and serum CRP level (cut-off value, 0.3 mg/dL; *P* = 0.005, Fig. 2c) as prognostic parameters for PFS, while there was a marginal difference between patients with low and those with normal serum sodium levels (*P* = 0.087; cut-off value, 135 mmol/L, Fig. 2e). In the univariate analysis for OS, the best objective response (*P* = 0.007), Heng risk score (*P* < 0.0001, Fig. 1d), serum CRP level (*P* = 0.012, Fig. 2d), and serum sodium level (*P* = 0.017, Fig. 2f) was associated with the OS. Multivariate analyses were performed for the following selected factors: the best objective response, serum CRP level, and serum sodium level. As a result, the best objective response was identified as a prognostic factor for PFS. The hazard ratios (HRs) for PFS of SD, PD, and NE patients were 1.67 [95 % confidence interval (CI) 0.619–4.60], 1.88 (0.52–6.84), and 3.56 (1.00–12.9), respectively, as compared to the CR/PR group. Similarly, the HRs for OS of SD, PD, and NE patients were 4.51 (95 % CI 0.95–21.4), 7.93 (1.36–47.8), and 4.88 (1.02–23.2), respectively, as compared to the CR/PR group (Table 4). The Heng risk score also seemed to dramatically stratify the patients, while serum CRP and sodium levels were not independent prognostic parameters. Patients with a favorable Heng score had a median PFS of 36.8 months, whereas the median PFS of those with intermediate and poor Heng scores was 9.8 and 2.7 months, respectively. In addition, the incidence of sunitinib related-hypertension was associated with a favorable tumor response to the drug.

**Table 4:** Cox proportional models for prognostic factors

Variables	Progression-free survival			Overall survival		
	HR	95 % CI	*P* value	HR	95 % CI	*P* value
Best objective response on target lesions						
CR/PR	1			1		
SD	1.67	0.61–4.60	0.23	4.51	0.95–21.4	0.06
PD	1.88	0.52–6.84	0.09	7.93	1.36–47.8	0.02
NE	3.56	1.00–12.9	0.05	4.88	1.02–23.2	0.04
Heng risk group						
Favorable	1			1		
Intermediate	2.70	0.97–7.56	0.06	2.69	0.61–11.9	0.19
Poor	14.50	4.42–48.0	0.001	10.1	2.03–50.6	0.01
Serum CRP level (mg/dL)						
≤0.3	1			1		
0.3<	1.68	0.72–3.96	0.23	1.50	0.58–3.91	0.40
Serum sodium level (mmol/L)						
≥135	NA			1		
135>				1.65	0.63–4.30	0.31
Hypertension related to sunitinib						
Yes	1			NA		
No	3.14	1.55–6.36	0.002			

*HR* hazard ratio; *CI* confience interval; *CR* complete response; *PR* pertial response; *SD* stable disease; *PD* progressive disease; *NE* not evaluable; *MSKCC* Memorial Sloan Kettering Cancer Center; *CRP* C-reactive protein; *NA* not analyzed

## Discussion

The era of molecular targeted therapies has identified several new agents with activity in mRCC. Many studies have been conducted since the introduction of molecular targeted therapies for mRCC in order to investigate the clinical relevance of tumor response [8–13]. Choi et al. [18] established a reliable and quantitative evaluation of tumor response using the change in Hounsfield unit on CT and ^18^F-fluorodeoxyglucose positron emission tomography in patients with gastrointestinal stromal tumors treated with imatinib. Though quite provocative, to date, the conventional RECIST criteria is utilized in most reports on mRCC, including the present study, because its ease and convenience to be applied in clinical setting. Although the association between tumor response with sunitinib and superior outcomes has been reported in many studies of European cohorts [8–13], there is a lack of such data in Japanese patients. Ethnic or geographic differences could lead to variation in the sensitivity and tolerance to drugs, resulting in discrepancies in treatment outcomes among patients of different races. Therefore, the present study was conducted to confirm the association between tumor response and the PFS or OS in Japanese patients with mRCC treated with sunitinib.

Our findings indicated that patients with CR/PR as their best objective response benefited the most from the treatment and achieved prolonged PFS and OS in comparison with others (Table 4). The results are supported by other reports on prolonged treatment duration in patients with CR or PR as the best objective response to targeted therapies [9–12]. In contrast, those with intrinsic drug resistance and/or poor tolerance to sunitinib, i.e., PD or NE, attained the worst outcomes. The substantial portion (68 %) of our cohort was categorized into the CR/PR or SD group (Table 1). In a recent study [19], mRCC patients receiving TKI treatment were stratified into several subgroups according to tumor shrinkage (−100 to −60 %, −59 to −30 %, and −29 to 0 % shrinkage), gain of tumor size (1–19 % and ≥20 %), or occurrence of new lesions, and treatment outcomes were compared among the groups. Abel et al. [9] reported that a 10 % early reduction of tumor size (defined as decrease within 60 days of treatment initiation) was a good cut-off value for OS prediction. Patient stratification according to gain or reduction in tumor size with more cut-off points might improve the prognostic value of tumor response evaluation during targeted therapy.

van der Veldt et al. [20] evaluated the tumor response in mRCC patients treated with sunitinib by using the Choi response criteria. They concluded that these criteria had a significantly better predictive value for the PFS and OS in patients with PR than the RECIST criteria. Furthermore, density measurements could provide additional predictive value, especially in patients without significant change in the tumor size. Previously, Smith et al. established two novel radiologic assessment tools to evaluate mRCC patients’ response to molecular targeted therapy [21]. The Size and Attenuation CT criteria and Morphology, Attenuation, Size, and Structure criteria using contrast enhancement on CT findings were proved better response assessment methods than the RECIST and modified Choi criteria [22].

The Memorial Sloan Kettering Cancer Center (MSKCC) model and Heng risk classification model are two major tools for prediction of prognosis and response to treatment in patients with mRCC [14, 23]. The main difference between the two is that they were developed predominantly for patients treated with cytokines and vascular endothelial growth factor-targeted agents. The Heng risk classification model has been validated in a large international multicenter dataset [24], and it is currently the most widely used model. The Heng risk classification was applied to our cohort because the patients were treated with sunitinib. In fact, our patients were evaluated using both the Heng and MSKCC models; however, the distribution of risk was almost the same (data not shown).

The incidence of adverse effects during targeted therapy and survival outcomes of mRCC patients have been previously reported [25, 26]. We retrospectively evaluated the influence of clinical toxicities on patients’ outcomes. Our findings suggested that sunitinib-related hypertension was a predictive factor associated with good tumor response and significantly longer PFS, but not OS, in mRCC patients (Tables 3, 4). The results are supported by previous studies reporting an association between hypertension induced by bevacizumab and increased PFS [27] and between axitinib-induced hypertension with increased OS [28]. Sire et al. [29] showed that the development of grade 2–4 hypertension was a favorable prognostic factor for mRCC patients treated with first- or second-line sunitinib, sorafenib, or bevacizumab. However, the potential of sunitinib-related toxicities, including hypertension and hypothyroidism, as predictive markers requires further validation in well-designed prospective clinical trials.

The retrospective nature of our study, participation by only five institutes, limited number of patients, and other residual confounding factors were potential sources of study bias. Additionally, the influence of subsequent therapies was not investigated in this patient cohort.

## Conclusions

The present study explored the best objective tumor response of targeted metastatic sites as a prognostic factor in mRCC. Our findings provided a rationale that good and durable response to sunitinib achievable in mRCC patients might be associated with an improved overall survival. Future studies are warranted to validate our findings. In addition, it is important for future clinical development to clearly define our end-points in clinical trials to determine the influence on clinical outcomes in patients with mRCC.

## Abbreviations

RECIST: Response Evaluation Criteria in Solid Tumors
CR: complete response
PR: partial response
SD: stable disease
PD: progressive disease
NE: not evaluated
mRCC: metastatic renal cell carcinoma
TKI: tyrosine kinase inhibitor
mTORI: mammalian targeted of rapamycin inhibitor
OS: overall survival
PFS: progression-free survival
ULN: upper limit of normal
CT: computed tomography
ECOG-PS: Eastern Cooperative Oncology Group performance status
CRP: C-reactive protein
HR: hazard ratio
CI: confidence interval

## References

[1] Wagstaff J (2007). Renal cell cancer: is immunotherapy dead?. Ann Oncol.

[2] Miyake M, Anai S, Fujimoto K (2012). 5-fluorouracil enhances the antitumor effect of sorafenib and sunitinib in a xenograft model of human renal cell carcinoma. Oncol Lett.

[3] Miyake M, Fujimoto K, Tanaka M (2009). Immunochemotherapy with interferon-α, interleukin-2, 5-fluorouracil, and cimetidine for patients with advanced renal cell carcinoma. J Nara Med Assoc.

[4] Escudier B, Albiges L, Blesius A (2010). How to select targeted therapy in renal cell cancer. Ann Oncol.

[5] Motzer RJ, Hutson TE, Tomczak P (2009). Overall survival and updated results for sunitinib compared with interferon alfa in patients with metastatic renal cell carcinoma. J Clin Oncol.

[6] Motzer RJ, Rini BI, Bukowski RM (2006). Sunitinib in patients with metastatic renal cell carcinoma. JAMA.

[7] Dutcher JP (2013). Recent developments in the treatment of renal cell carcinoma. Ther Adv Urol.

[8] Seidel C, Busch J, Weikert S (2012). Progression free survival of first line vascular endothelial growth factor-targeted therapy is an important prognostic parameter in patients with metastatic renal cell carcinoma. Eur J Cancer.

[9] Abel EJ, Culp SH, Tannir NM (2011). Early primary tumor size reduction is an independent predictor of improved overall survival in metastatic renal cell carcinoma patients treated with sunitinib. Eur Urol.

[10] Jain RK, Lee JJ, Ng C (2012). Change in tumor size by RECIST correlates linearly with overall survival in phase I oncology studies. J Clin Oncol.

[11] Molina AM, Zhang J, Lin X (2012). Sunitinib objective response (OR) in metastatic renal cell carcinoma (mRCC): analysis of 1,059 patients treated on clinical trials. ASCO Meet Abstr.

[12] Grunwald V, Seidel C, Fenner M, et al. Use of early tumor shrinkage as a response to VEGF inhibitors as a predictor of progression-free survival (PFS) and overall survival (OS) in patients with metastatic renal cell carcinoma (mRCC). J Clin Oncol. 2012;30 Suppl:abstract 4631.

[13] Busch J, Seidel C, Goranova I (2014). Categories of response to first line vascular endothelial growth factor receptor targeted therapy and overall survival in patients with metastatic renal cell carcinoma. Eur J Cancer.

[14] Heng DY, Xie W, Regan MM (2009). Prognostic factors for overall survival in patients with metastatic renal cell carcinoma treated with vascular endothelial growth factor-targeted agents: results from a large, multicenter study. J Clin Oncol.

[15] Naito S, Yamamoto N, Takayama T (2010). Prognosis of Japanese metastatic renal cell carcinoma patients in the cytokine era: a cooperative group report of 1463 patients. Eur Urol.

[16] Schutz FA, Xie W, Donskov F (2014). The impact of low serum sodium on treatment outcome of targeted therapy in metastatic renal cell carcinoma: results from the International Metastatic Renal Cell Cancer Database Consortium. Eur Urol.

[17] Eisenhauer EA, Therasse P, Bogaerts J (2009). New response evaluation criteria in solid tumours: revised RECIST guideline (version 1.1). Eur J Cancer.

[18] Choi H, Charnsangavej C, Faria SC (2007). Correlation of computed tomography and positron emission tomography in patients with metastatic gastrointestinal stromal tumor treated at a single institution with imatinib mesylate: proposal of new computed tomography response criteria. J Clin Oncol.

[19] Seidel C, Busch J, Weikert S (2013). Tumour shrinkage measured with first treatment evaluation under VEGF-targeted therapy as prognostic marker in metastatic renal cell carcinoma (mRCC). Br J Cancer.

[20] van der Veldt AA, Meijerink MR, van den Eertwegh AJ (2010). Choi response criteria for early prediction of clinical outcome in patients with metastatic renal cell cancer treated with sunitinib. Br J Cancer.

[21] Smith AD, Lieber ML, Shah SN (2010). Assessing tumor response and detecting recurrence in metastatic renal cell carcinoma on targeted therapy: importance of size and attenuation on contrast-enhanced CT. AJR Am J Roentgenol.

[22] Smith AD, Shah SN, Rini BI (2010). Morphology, Attenuation, Size, and Structure (MASS) criteria: assessing response and predicting clinical outcome in metastatic renal cell carcinoma on antiangiogenic targeted therapy. AJR Am J Roentgenol.

[23] Motzer RJ, Bacik J, Murphy BA (2002). Interferon-alfa as a comparative treatment for clinical trials of new therapies against advanced renal cell carcinoma. J Clin Oncol.

[24] Heng DY, Xie W, Regan MM (2013). External validation and comparison with other models of the International Metastatic Renal-Cell Carcinoma Database Consortium prognostic model: a population-based study. Lancet Oncol.

[25] Szmit S, Langiewicz P, Złnierek J (2012). Hypertension as a predictive factor for survival outcomes in patients with metastatic renal cell carcinoma treated with sunitinib after progression on cytokines. Kidney Blood Press Res.

[26] Di Fiore F, Rigal O, Ménager C (2011). Severe clinical toxicities are correlated with survival in patients with advanced renal cell carcinoma treated with sunitinib and sorafenib. Br J Cancer.

[27] Bono P, Elfving H, Utriainen T (2009). Hypertension and clinical benefit of bevacizumab in the treatment of advanced renal cell carcinoma. Ann Oncol.

[28] Rini BI, Schiller JH, Fruehauf JP, et al. Association of diastolic blood pressure 190 mm Hg with overall survival in patients treated with axitinib. J Clin Oncol. 2008; 26:15S, abstr 3543.

[29] Sire M, Wallerand H, Kilkoski F (2008). Anti-angiogenic treatment in the management of metastatic renal cell carcinoma. Bull Cancer.

